# Faculty diversity trends in physical medicine and rehabilitation by gender, race, and ethnicity in the United States, 1977–2021

**DOI:** 10.1002/pmrj.13291

**Published:** 2024-12-24

**Authors:** Jad Lawand, Leena Mazhar, Ali Rauf, Jeffrey Ding, Javed Siddiqi, Sabeen Tiwana, Naznin Virji‐Babul, Faisal Khosa

**Affiliations:** ^1^ University of Texas Medical Branch John Sealy School of Medicine Galveston Texas USA; ^2^ Faculty of Medicine University of British Columbia Vancouver British Columbia Canada; ^3^ University of North Texas Health Science Center Texas College of Osteopathic Medicine Fort Worth Texas USA; ^4^ Department of Neurosurgery Arrowhead Regional Medical Center Colton California USA; ^5^ Faculty of Dentistry University of British Columbia Vancouver British Columbia Canada

## Abstract

**Background:**

This study describes the gender and racial/ethnic trends in academic physical medicine and rehabilitation (PM&R) and the shifts that have taken place in more than 4 decades.

**Objective:**

To gauge the diversity in gender and race/ethnicity across academic degrees, academic ranks, chair positions, and tenure status in the academic workforce of PM&R.

**Design:**

Surveillance study.

**Setting and Methods:**

The data for academic PM&R faculty were self‐reported and obtained from the annual Faculty Roster report of the Association of American Medical Colleges from 1977 to 2021.

**Main Outcome Measures:**

To compare the distribution of academic degree, rank, chair position, and tenure status over time, the percentage composition for each category was calculated for a period of 45 years. The temporal trends were depicted by plotting the counts and proportion changes, and the progress in terms of racial representation was illustrated by graphing the absolute changes in the percentage composition.

**Results:**

Despite an overall increase in the representation of women, women remained underrepresented in the full professor rank in 2021, at only 32.1% of full professors. The instructor category was the only category in which the proportion of women faculty was higher in 2021 (62.8%) than in 1977 (58.5%). Asian faculty had the greatest increase in representation at all ranks, with the proportion of Asian full professors increasing from 1.8% to 11.4%, associate professors increasing from 7.4% to 14.4%, and assistant professors increasing from 11.2% to 20.2%. Women's representation as department chairs increased from 12.5% to 23.7% and Asians from 2.5% to 15.3%.

**Conclusion:**

Overall, although there was an increase in the number of women and underrepresented minority faculty in academic PM&R over the study period, disparities based on gender and ethnicity/race persisted, particularly in higher academic ranks and leadership positions.

## INTRODUCTION

By 2050, racial and ethnic minorities are anticipated to make up the bulk of the population. Today, 14% and 5% of the U.S. population includes Hispanics and Asians, respectively.^1^ By 2050, Hispanics and Asians will make up 29% and 9% of U.S. population, respectively.^1^ Projections reveal that by the year 2050 roughly one in five Americans will be foreign born.^1^

Medical schools have adopted holistic admissions processes and invested in increasing qualified applicants from marginalized backgrounds.^2^ Even though marginalized groups may soon be the majority, there is still a lack of diversity among practicing physicians. Men and White physicians comprise 56.2% and 64.1% of the physician workforce, respectively.^2^ A similar divergence exists within academic medicine. A 2018 study revealed that women were less likely to receive the rank of professor and remain in academic medical careers than their male counterparts.^3^ Regarding medical school admissions, applicants who identify as Black have lagged behind other groups,^2^ despite the growth of the Black population within the United States.

Specifically, within the discipline of physical medicine and rehabilitation (PM&R), there is a similar discrepancy. Over the past 48 years, women have received only 15.9% of awards presented by the American Academy of Physical Medicine and Rehabilitation.^4^ Furthermore, disparities are also evident in the development of clinical practice guidelines, where both gender and racial/ethnic diversity are lacking. For instance, a study by Verduzco‐Gutierrez et al. examined author diversity on clinical practice guideline committees, revealing significant gaps in representation. This study highlights the ongoing challenges in ensuring diversity and equity within the academic PM&R community.

Moreover, non‐White PM&R faculty were found to occupy more assistant than full professor positions at U.S. medical schools.^5^ This indicates that there is a potential to increase diversity within medicine, specifically PM&R.^6^

Although our group hypothesized an increase in diversity within PM&R over the last 4 decades, there is minimal literature supporting this. One study looking at data from 2007 to 2018 found that men faculty held at least 75% of full professor positions at any time in the given period and Asian faculty saw the greatest increase in proportion of full professors (3.7% to 10%).^7^ In the following study, our intention was to expand the current knowledge on diversity in PM&R by analyzing trends in gender and race as well as ethnic diversity in chair position, academic rank, degree, and tenure status within PM&R over a 45‐year period (1977–2021).

## METHODOLOGY

The study obtained self‐reported data for PM&R from the Association of American Medical Colleges (AAMC) annual Faculty Roster report spanning 45 years from 1977 to 2021, including race/ethnicity, gender, academic degree, academic rank, chair position, and tenure status.

Acknowledging the spectrum of gender, it is worth noting that the data provided by the AAMC were presented in binary categories labeled as “sex,” delineated by “female” and “male.” In line with the original terminology, we opted to maintain consistency in our report. Additionally, we made sure to honor the language used in the cited literature, employing the terms “woman” and “man” when referenced, as per the original source. The data for the study were obtained without any omission and the methodology used has been validated in several recent publications.^7^, ^8^, ^9^ The race/ethnicity categories included White, Asian, Hispanic/Latino, Black/African American, Multiple race, Other, and Unknown. Academic degree was categorized as MD, PhD, or other doctoral degree; MD/PhD or PhD or MD with other doctoral degree; and other. Academic rank was categorized as full professor, associate professor, assistant professor, instructor, chair, and other degree type. Tenure tracks were categorized as tenured, on track, and not on track.

The study aimed to analyze gender and race/ethnicity distribution in the categories of academic degree, academic rank, and tenure status from 1977 to 2021. The percentage composition for each category was calculated over the 45‐year period to compare the overall distribution, and proportion changes were plotted to show the temporal trends. The study also graphed the absolute changes in racial percentage makeup of each category over the study period to highlight any progress.

The Behavioral Research Ethics Board of the institute deemed this study exempt from Research Ethics Board review because the data analyzed are publicly available and the publication of the results does not create new identifiable information.

## RESULTS

### Academic degree

During the 45‐year study period, the total number of faculty in academic PM&R increased by 1264, with the majority of the increase occurring among White faculty members, who saw an increase of 663 persons, and with a significant increase in the number of female faculty members as well (Table 1). In terms of degree categories, most of the increase in the MD and PhD categories was among White faculty members, followed by Asian faculty members. However, there were also increases in faculty members of other non‐White groups, such as Black/African American, Hispanic/Latino, and Multiple races. Black/African American faculty members increased from 1.6% to 4.2% over the 45‐year span studied, whereas Hispanic/Latino faculty members increased from 2.1% to 4.1%.

**TABLE 1:** Gender and race/ethnicity makeup and the absolute changes in academic degrees between 1977 and 2021.

Degree		White	Asian	Black/African American	Hispanic/Latino	Multiple race	Unknown	Others	Total	Male	Female
All degrees	1977	462 (82.4%)	47 (8.4%)	9 (1.6%)	12 (2.1%)	6 (1.1%)	25 (4.5%)	0 (0%)	561	383 (68.2%)	178 (31.7%)
	2021	1125 (61.6%)	316 (17.3%)	76 (4.2%)	74 (4.1%)	64 (3.5%)	149 (8.2%)	21 (1.2%)	1825	932 (51.1%)	893 (48.9%)
	Absolute change	663	269	67	62	58	124	21	1264	549	715
MD only	1977	219 (74.2)	43 (14.6%)	5 (1.7%)	10 (3.4%)	5 (1.7%)	13 (4.4%)	0 (0.0%)	295	231 (78.3%)	64 (21.7%)
	2021	702 (60.8%)	262 (22.7)	57 (4.9%)	60 (5.2%)	47 (4.1%)	13 (1.1%)	13 (1.1%)	1154	646 (56.0%)	508 (44.0%)
	Absolute change	483	219	52	50	42	0	13	859	415	444
PhD or other	1977	100 (93.5%)	2 (1.9%)	0 (0%)	0 (0%)	1 (0.9%)	4 (3.7%)	0 (0.0%)	107	83 (77.6%)	24 (22.4%)
Doctoral degree	2021	314 (64.1%)	37 (7.6%)	12 (2.4%)	5 (1.0%)	11 (2.2%)	107 (21.8%)	4 (0.8%)	490	207 (42.2%)	283 (57.7%)
	Absolute change	214	35	12	5	10	103	4	383	124	259
MD and PhD or	1977	13 (86.7%)	1 (6.7%)	0 (0.0%)	0 (0.0%)	0 (0.0%)	1 (6.7%)	0 (0.0%)	15	13 (86%)	2 (13.3%)
MD and other	2021	45 (66.2%)	11 (16.2%)	5 (7.4%)	3 (4.4%)	1 (1.5%)	2 (2.9%)	1 (1.5%)	68	42 (61.8%)	26 (38.2%)
Doctoral degree	Absolute change	32	10	5	3	1	1	1	53	29	24
Other	1977	89 (89.9%)	1 (1.0%)	3 (3%)	1 (1.0%)	0 (0.0%)	5 (5.1%)	0 (0.0%)	99	32 (32.3%)	67 (67.7%)
	2021	32 (51.6%)	1 (1.6%)	0 (0.0%)	4 (6.5%)	2 (3.2%)	21 (33.9%)	2 (3.2%)	62	20 (32.3%)	42 (67.7%)
	Absolute change	−57	0	−3	3	2	16	2	−37	−12	−25
Unknown	1977	41 (91.1%)	0 (0.0%)	1 (2.2%)	1 (2.2%)	0 (0.0%)	2 (4.4%)	0 (0.0%)	45	24 (53.3%)	21 (46.7%)
	2021	32 (62.7%)	5 (9.8%)	2 (3.9)	2 (3.9%)	3 (5.9%)	6 (11.8%)	1 (2.0)	51	17 (32.3%)	34 (66.7%)
	Absolute change	−9	5	1	1	3	4	1	6	−7	13

There were significant increases in the number of female faculty members compared to male faculty members, with an increase of 715 and 549, respectively. However, male faculty members still had a higher overall absolute number of faculty members and were more prevalent in all degree categories except for the PhD or other doctoral degree category.

**FIGURE 1:**
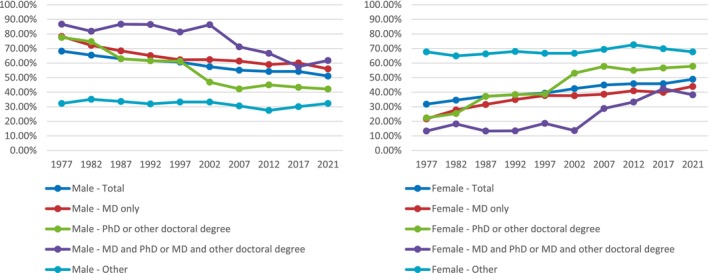
Changes in the percentage makeup of academic degrees by gender from 1977 to 2021.

White faculty members had the highest percentage in all degree categories, although there was an overall decrease from 82.4% in 1977 to 61.6% in 2018, with the largest increase during this period of Asian and Unknown categories. A closer examination of changes by gender in the percentage makeup of degrees over the study period revealed a steady increase in females' percentages in all categories, except for MD and PhD or MD and other doctoral degree categories (Figure 1). Female faculty members occupy an increasing percentage in this category, from 13.3% in 1977 to 38.2% in 2018.

### Academic rank

The number of all academic faculty increased by 1264 between 1977 and 2021. There was an increase in total number of faculty for all academic ranks, except for instructors, with a decrease of 14 (Table 2). The greatest increase in total number of faculty was at the assistant professor level. In all categories except instructors, White faculty demonstrated the greatest increase in absolute number among all races and ethnicities (663 faculty), with Asian the second greatest increase (269 faculty). Nonetheless, similar to trends seen in academic degrees, the proportion of total White faculty amid the total sample of this study decreased in all categories (82.4% in 1977 to 61.6% in 2021), with the greatest percentage increase in Asian full professors (from 1.8% to 11.4% of full professors), Asian assistant professors (from 11.2% to 20.2%), and Asian associate professors (7.4% to 14.4%). Universally, proportions of Black/African American, Hispanic/Latino, Multiple Race, Unknown, and Other faculty increased among full professors, associate professors, assistant professors, and instructors.

**TABLE 2:** Gender and race/ethnicity makeup and the absolute changes in academic rank between 1977 and 2021.

Degree		White	Asian	Black/African American	Hispanic/Latino	Multiple race	Unknown	Others	Total	Male	Female
All faculty	1977	462 (82.4%)	47 (8.4%)	9 (1.6)	12 (2.1%)	6 (0.4%)	25 (4.5%)	0 (0.0%)	561	383 (68.3%)	178 (31.7%)
	2021	1125 (61.6%)	316 (17.3%)	76 (4.2%)	74 (4.1%)	64 (3.5%)	149 (8.2%)	21 (1.2%)	1825	932 (51.1%)	893 (48.9%)
	Absolute change	663	269	67	62	58	124	21	1264	549	715
Professors	1977	100 (91.7%)	2 (1.8%)	1 (0.9%)	1 (0.9%)	1 (0.8%)	4 (3.7%)	0 (0.0%)	109	99 (90.8%)	10 (9.2%)
	2021	183 (74.4%)	28 (11.4%)	5 (2.0%)	9 (3.7%)	7 (2.8%)	14 (5.7%)	0 (0.0%)	246	167 (67.9%)	79 (32.1%)
	Absolute change	83	26	4	8	6	10	0	137	68	69
Associate	1977	91 (84.3%)	8 (7.4%)	2 (1.9%)	2 (1.9%)	2 (1.7%)	3 (2.8%)	0 (0.0%)	108	81 (75%)	27 (25%)
Professor	2021	229 (65.8%)	50 (14.4%)	10 (2.9%)	22 (6.3%)	8 (2.3%)	24 (6.9%)	5 (1.5%)	348	170 (48.9%)	178 (51.1%)
	Absolute change	138	42	8	20	6	21	5	240	89	151
Assistant	1977	151 (77.0%)	22 (11.2%)	2 (1.9%)	6 (3.1%)	3 (1.6%)	12 (6.1%)	0 (0.0%)	196	140 (71.4%)	56 (28.6%)
Professor	2021	628 (57.2%)	222 (20.2%)	58 (5.3%)	36 (3.3%)	44 (4.0%)	98 (8.9%)	11 (1.0%)	1097	543 (49.5%)	554 (50.5%)
	Absolute change	477	200	56	30	41	86	11	901	403	498
Instructor	1977	107 (79.3%)	15 (11.1%)	4 (3.0%)	3 (2.2%)	0 (1.6%)	6 (4.4%)	0 (0.0%)	135	56 (41.5%)	79 (58.5%)
	2021	76 (62.8%)	16 (13.2%)	3 (2.5%)	5 (4.1%)	5 (4.1%)	11 (9.1%)	5 (4.1%)	121	45 (37.2%)	76 (62.8%)
	Absolute change	−31	1	−1	2	5	5	5	−14	−11	−3
Other	1977	13 (100%)	0 (0.0%)	0 (0.0%)	0 (0.0%)	0 (0.0%)	0 (0.0%)	0 (0.0%)	13	7 (53.8%)	6 (46.2%)
	2021	9 (69.2%)	0 (0.0%)	0 (0.0%)	2 (15.4%)	0 (0.0%)	2 (15.4%)	0 (0.0%)	13	7 (53.8%)	6 (46.2%)
	Absolute change	−4	0	0	2	0	2	0	0	0	0

**FIGURE 2:**
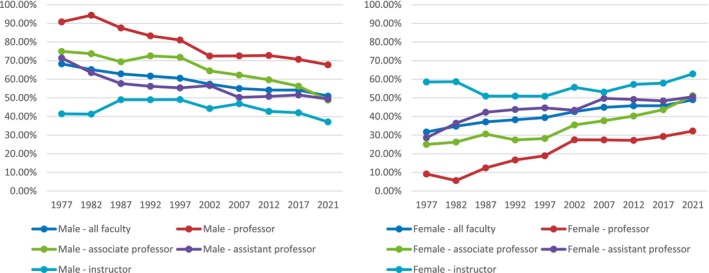
Changes in the percentage makeup of academic ranks by gender from 1977 to 2021.

There was a higher increase in total number of female faculty as full professors, associate professors, and assistant professors, whereas there was a net decrease in both male and female instructors (with an absolute decrease of 11 male instructors and 3 female instructors). There remains a higher total number of male full professors (67.9% male versus 32.1% female). The total number of male associate professors and assistant professors is approximately equal to the number of female associate professors and assistant professors (170 male versus 178 female associate professors; 543 male versus 553 female assistant professors). Instructor is the only category with a higher total number of female faculty, both in 1977 and in 2021. Figure 2 compares the changes over 45 years in the percentage makeup of each academic rank by gender.

### Chair position

The total number of chairpersons increased from 40 in 1977 to 59 in 2021. Over 45 years, the percentage of White chairpersons decreased (90% in 1977 vs. 72.9% in 2021), while the percentage of Asian, Black, Hispanic/Latino, and Multiple Race faculty increased. The total number of male chairpersons increased (35 in 1977 vs. 45 in 2021) but the proportion that was male decreased (87.5% in 1977 vs. 76.3% in 2021). Conversely, the total number of female chairpersons increased (5 in 1977 vs. 14 in 2021) and the overall proportion increased (12.5% in 1977 vs. 23.7% in 2021) (Table 3).

**TABLE 3:** Yearly percentages and changes between 1997 and 2021 of academic medicine leaders by gender and race/ethnicity.

Chairperson	1977	1982	1987	1992	1997	2002	2007	2012	2017	2021
White	36 (90%)*	29 (90.6%)*	39 (88.6%)*	43 (82.7%)*	45 (84.9%)*	42 (79.2%)*	42 (79.2%)*	45 (72.6%)*	48 (78.7%)*	43 (72.9%)*
Asian	1 (2.5%)	2 (6.3%)	2 (4.5%)	4 (7.7%)	4 (7.5%)	5 (9.4%)	5 (9.4%)	5 (8.1%)	5 (8.2%)	9 (15.3%)
Black/African American	1 (2.5%)	1 (3.1%)	2 (4.5%)	2 (3.8%)	1 (1.9%)	1 (1.9%)	2 (3.8%)	2 (3.2%)	2 (3.3%)	3 (5.1%)
Hispanic/Latino	0 (0.0%)	0 (0.0%)	1 (2.3%)	1 (1.9%)	2 (3.8%)	2 (3.8%)	2 (3.8%)	6 (9.7%)	3 (4.9%)	1 (1.7%)
Multiple race	0 (0.0%)	0 (0.0%)	0	0	0	1 (1.9%)	1 (1.9%)	3 (4.8%)	3 (4.9%)	3 (5.1%)
Unknown	2 (5.0%)	0 (0.0%)	0	1 (1.9%)	0	0	0	1 (1.6%)	0 (0.0%)	0 (0.0%)
Others	0 (0.0%)	0 (0.0%)	0	1 (1.9%)	1 (1.9%)	2 (3.8%)	1 (1.9%)	0 (0.0%)	0 (0.0%)	0 (0.0%)
Total	40	32	44	52	53	53	53	62	61	59
Female	5 (12.5%)	3 (9.4%)	4 (9.1%)	7 (13.5%)	6 (11.3%)	10 (18.9%)	10 (18.9%)	8 (18.0%)	11 (18.0%)	14 (23.7%)
Male	35 (87.5%)	29 (90.6%)	40 (90.9)	45 (86.5%)	47 (88.7%)	43 (81.1%)	43 (81.1%)	54 (87.1%)	50 (82.0%)	45 (76.3%)

* Percentage only.

### Tenure status

**FIGURE 3:**
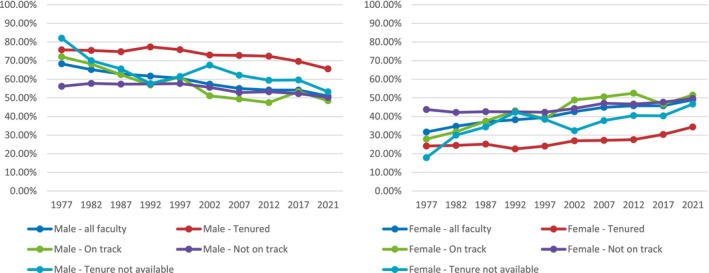
Changes in percentage makeup of tenure status by gender from 1977 to 2021.

During the 45‐year study period, the total number of tenured faculty decreased (130 in 1977 versus 82 in 2021), whereas the number of faculty on a tenure track increased (104 in 1977 and 167 in 2021) and the number of faculty not on a tenure track also increased (143 in 1977 vs. 827 in 2021) (Table 4). The overall percentage of tenured faculty has decreased drastically, ranging from 25.8% of faculty in 1977 to 5.6% of faculty in 2021. The percentage of on‐track faculty also decreased, ranging from 21.9% of faculty in 1977 to 15.6% of faculty in 2021. Among all those tenured, the White faculty had the highest percentage, varying between 89.7%% in 1977 and 80.4% in 2021, and male faculty had more than 50% in all categories except tenure track. Faculty who identified as Asian showed an increase in proportion of tenured faculty, on‐track faculty, and not‐on‐track faculty. Faculty who identified as Black/African American showed a decrease in proportion of tenured faculty (2.8% in 1977 vs. 2.0% in 2021) but showed an increase in proportion of on‐track faculty and not‐on‐track faculty. Over the 45‐year period, male faculty showed an overall trend of decreasing proportions in all tenure statuses whereas female faculty showed an overall trend of increasing proportions in all tenure statuses (Figure 3).

**TABLE 4:** Gender and ethnicity/race makeup and the absolute changes in tenure status between 1977 and 2021.

Academic levels		White	Asian	Black/African American	Hispanic/Latino	Multiple race	Unknown	Others	Total	Male	Female
All faculty	1977	462 (82.4%)	47 (8.4%)	9 (1.6%)	12 (2.1%)	6 (1.1%)	25 (4.5%)	0 (0.0%)	561	383 (68.3%)	178 (31.7%)
	2021	1125 (61.6%)	316 (17.3%)	76 (4.2%)	74 (4.1%)	64 (3.5%)	149 (8.2%)	21 (1.2%)	1825	932 (51.1%)	893 (48.9%)
	Absolute change	663	269	67	62	58	124	21	1264	549	715
Tenured	1977	130 (89.7%)	4 (2.8%)	4 (2.8%)	0 (0.0%)	3 (2.1%)	4 (2.8%)	0 (0.0%)	145	110 (75.9%)	35 (24.1%)
	2021	82 (80.4%)	10 (9.8%)	2 (2.0%)	3 (2.9%)	4 (3.9%)	1 (1.0%)	0 (0.0%)	102	67 (65.7%(	35 (34.3%)
	Absolute change	−48	6	−2	3	1	−3	0	−43	−43	0
On track	1977	104 (84.6%)	13 (10.6%)	1 (0.8%)	7 (5.7%)	3 (2.4%)	1 (0.8%)	0 (0.0%)	123	93 (75.6%)	36 (29.3%)
	2021	167 (58.7%)	51 (18.6%)	6 (2.1%)	15 (5.3%)	9 (3.2%)	33 (11.6%)	3 (1.1%)	284	138 (48.6%)	146 (51.4%)
	Absolute change	63	38	5	8	6	32	3	161	45	110
Not on track	1977	143 (84.6%)	19 (11.2%)	2 (1.2%)	1 (0.6%)	0 (0.0%)	4 (2.4%)	0 (0.0%)	169	95 (56.2%)	74 (43.8%)
	2021	827 (61.4%)	234 (17.4%)	66 (4.9%)	40 (3.0%)	49 (3.6%)	115 (8.5%)	17 (1.3%)	1348	679 (50.4%)	669 (46.7%)
	Absolute change	684	215	64	39	49	111	17	1179	584	595
Tenure not available	1977	14 (50.0%)	2 (7.1%)	1 (3.6%)	0 (0.0%)	0 (0.0%)	11 (39.3%)	0 (0.0%)	28	23 (82.1%)	5 (17.9%)
	2021	49 (54.4%)	20 (22.2%)	2 (2.2%)	16 (17.8%)	2 (3.2%)	0 (0.0%)	1 (1.1%)	90	48 (53.3%)	42 (46.7%)
	Absolute change	35	18	1	16	2	−11	1	62	25	37
Unknown	1977	71 (78.9%)	9 (10.0%)	1 (1.1%)	4 (4.4%)	0 (0.0%)	5 (5.6%)	0 (0.0%)	90	62 (68.9%)	28 (31.1%)
	2021	0 (0.0%)	1 (100%)	0 (0.0%)	0 (0.0%)	0 (0.0%)	0 (0.0%)	0 (0.0%)	1	0 (0.0%)	1 (100%)
	Absolute change	−71	−8	−1	−4	0	−5	0	−89	−62	−27

## DISCUSSION

Between the consecutive years from 1977 and 2021, the gender and racial/ethnic disparities persisted in academic PM&R faculty. The study indicated that there was a greater increase in the number of women faculty across all degree levels in academic PM&R. Despite an increase in women's representation at all academic levels, the largest disparity remained at the professor level.

Disparities persisted among non‐White racial/ethnic minorities, with the study discovering that faculty identifying as Asian showed the largest increase, whereas those identifying as Black/African American and Hispanic showed lesser amounts of change. Additionally, although both gender and ethnicity/race disparities declined over the 45‐year period, the study showed that the highest gaps continued to be in the higher academic ranks and leadership positions.

The overall percentage of women faculty increased from 31.7% in 1977 to 48.9% in 2021, indicating an increased proportion of women's representation in PM&R faculty. Although women make up over half of the associate professor and assistant professor positions, there remains a gender gap in the highest academic position of professor and the chairperson leadership level, with women accounting for only 32.1% and 23.7% of these positions, respectively. Over the 45‐year period, there was a gradual increase in the percentage of women faculty in both professor and chairperson positions, reaching the highest proportion in women positions in 2021. The study also highlighted an increase in the proportion of women in the instructor position, from 58.5% to 62.8% by the end of the analyzed period. Interestingly, despite more women faculty obtaining higher degrees, with 44% of faculty with an MD degree and 57.7% of faculty with a PhD or other doctoral degree being women, the gender gap in the highest academic and leadership positions persists. Studies from the AAMC show that as of 2019, women comprise just over 50% of all medical students,^10^ compared to only 24.9% in 1980,^2^ a trend that is consistent with the overall increase in degree status among women the field of PM&R. Women comprised only 34.3% of tenured faculty, despite making up over 50% of on‐track faculty. A study conducted by Edmunds et al. suggests that women may shy away from pursuing a career in academic medicine for various reasons, such as a greater inclination toward teaching over research, inadequate access to mentors or role models, and facing gender discrimination and bias in their medical profession.^11^ These multifactorial issues may contribute to the gender differences prevalent in PM&R and other medical fields.

Although the gap between White and non‐White faculty decreased since 1977, a large disparity still appeared between the two groups, as the majority of degree holders, faculty members, and chair members identified as White. Of all non‐White faculty, Asians had the highest percentage increase in degree, faculty, academic level, and chairperson position over the study period. Although those identifying as Black/African American and Hispanic/Latino faculty showed increased representation throughout most levels, the improvement was minimal. Of note, in 2021, out of the 59 total chairperson position, 9 were Asian, 3 were Black/African American, 1 was Hispanic, and 3 identified as multiple races. According to a study from Sanchez et al. that studied the diversity in academic PM&R versus other specialties, PM&R had a large disparity in representation of underrepresented minorities.^7^, ^12^ The majority of the highest positioned individuals in PM&R identified as White, followed by Asians, Hispanics, and Black/African Americans.^12^ An additional study from Odei et al. suggested a similar problem across other specialties, as the study from 1980 to 2019 revealed that the proportions of Asian, Black, and Hispanic individuals among faculty, senior faculty, and department chairs were low compared to their White counterparts.^13^ Asians were represented equitably as department chairs in otolaryngology, psychiatry, and dermatology, whereas Black/African American individuals were overrepresented only in family medicine.^13^, ^14^, ^15^, ^16^, ^17^, ^18^, ^19^, ^20^ A study from Samuel et al. analyzing internal medicine faculty and department chairs revealed that Asians comprised approximately 20% of total faculty, yet only 7% of department chairs.^21^ However, this was still the proportion of both Black/African American and Hispanic/Latino in the same positions.^21^ Our study also concluded that Asian faculty had the greatest proportional increase in professor and chairperson positions by the end of the study period. Faculty identifying as Asian made up 15.3% of total chairperson positions by 2021, the highest recorded number throughout the study period. Despite a gradual increase of total chairperson positions throughout the 45‐year period, the greatest number of faculty identifying as Black/African American for the chairperson position was three in 2021. For faculty identifying as Hispanic/Latino, the greatest total chairperson spots occupied in 1 year was six in 2012; however, the total had decreased to one in 2021. Although the data suggest that Asian faculty are beginning to be represented in more leadership positions in all medical fields, including PM&R, there remains a large gap between all underrepresented minority (URiM) faculty and their White and Asian counterparts, suggesting deeper systemic concerns that need to be addressed to bridge these gaps.

Several of these gaps in health care can be addressed by observing trends from a broader lens and understanding the reasoning behind why these gaps occur. Zhang et al. suggested that the basis of these gaps for Asians in PM&R leadership is in part attributable to stereotypes, and in agreement with this paper, found that faculty who identify as Asian obtain full professor or department chair level proportionally lower, although there has been a considerable increase over the past 20 years.^7^ Similarly, in terms of gender gaps, stereotypes and biases play detrimental roles in the progression of a woman's career in the workplace, which has been reflected in findings across industries and health care specialties. According to the World Health Organization, women comprise almost 70% of health and social care workers globally and yet it is estimated that they hold only around 25% of leadership roles in health care.^22^ A study from McKinsey showed that as of 2023, only 32% of C‐suite positions are held by women, and only 4% are women of color. When comparing this statistic to all other industries, 26% of C‐suite positions are held by women.^22^ The same study also reported that women are promoted to higher positions less often than men in the health care industry.^23^ Regarding PM&R, Dixon et al. reported that between the years of 2014 and 2022, 36%–39% of PM&R residents were women, whereas 47%–49% of residents from 11 other specialties were women.^24^ Other self‐reported data in PM&R from 2007 to 2018 suggested that while the increase in faculty members for both male and females was roughly equal, the number of females faculty decreased as academic level increased.^7^ Outside of PM&R, female faculty showed the same patterns of underrepresentation at higher ranks despite equal faculty members in various departments including microbiology, internal medicine, emergency medicine, public health, neurology, dermatology, obstetrics and gynecology, psychiatry, radiology, and academic medicine faculty from 2007 to 2018.^8^, ^9^, ^14^, ^15^, ^24^, ^25^, ^26^, ^27^, ^28^, ^29^ This stark trend across specialties is in accordance with present findings, highlighting a need for addressing such gaps.

Such trends can also be attributed to obstacles that women face in the workplace not only in health care but also in other industries. For example, a study from Benson et al. showed that female employees in retail received higher performance ratings than their male counterparts, yet received lower “potential” ratings and fewer promotions than their male counterparts.^30^ Women may also struggle to reach higher positions due to difficulties with obtaining higher credentials due to biases related to receiving awards. For example, a study by Silver et al. showed that in the field of PM&R from 1968 to 2015, The American Academy of Physical Medicine and Rehabilitation presented only 42 of the total 264 recognition awards to women, which was a lower proportion of the total amount of women in the field.^4^ Although a study from Gerull et al. regarding orthopedic surgery societies revealed that the number of awards given to women is proportionate to their involvement in the field, women were still less likely to receive leadership awards and less likely to receive awards in unblinded processes, reinforcing the biases already present in the field.^31^

URiM faculty also encounter comparable obstacles in obtaining awards and recognition, which can limit their opportunities to distinguish themselves with a more comprehensive curriculum vitae. Ginther et al. revealed that Black applicants had a 10% lower chance of receiving National Institutes of Health research funding even after controlling for other variables.^32^ Similarly, Boatright et al. discovered that Black and Asian medical students were less likely to be chosen for the Alpha Omega Alpha medical honor society.^33^ These discrepancies between racial minorities and their White counterparts could contribute to the challenges they face in advancing their careers.

It is important for various medical institutions and leaders, not just in PM&R, but in other fields as well, to investigate the reasons behind the persistence of gaps in diversity and inclusivity. By doing so, they can identify potential solutions and implement changes that will enhance their respective fields. For example, Escalon et al. suggest strategies including holistic review, implicit bias training, structured interviews, and targeted outreach toward underrepresented populations in PM&R to encourage student entry into the specialty.^34^ It is hoped that these efforts will result in a more comprehensive and prosperous field of physical medicine and rehabilitation that can positively influence trends in health care representation for future generations.

A bibliometric analysis of the highly cited publications on leadership revealed that research on succession planning in leadership was conspicuously absent.^33^ This lack of research and transparency may be the reason for the lack of women and minorities in senior academic ranks and leadership positions.^35^

### Limitation

Our study assumed a generalized definition of each racial/ethnic group (eg, Black faculty include those who identify as African American or other ancestry; Asians include those who identify as Chinese, Vietnamese, Indian, etc.), which prevents accurate portrayal of each racial subgroup's faculty composition. The study did not examine the combined effects of gender and race/ethnicity. Furthermore, only data for full‐time faculty were analyzed. Finally, our study does not conclude any objective correlation between the academic variables examined and the cause of underrepresentation of women and URiM.

## CONCLUSION

Over the 45‐year period between 1977 and 2021, women and URiM individuals have seen paramount increases in both the number and proportion of PM&R faculty at most academic levels, degrees, and chairperson statuses. Despite the sizeable increases, there continue to be disparities compared with men faculty and White faculty. The addition of data for part‐time faculty may partially illustrate a cause for underrepresentation of women and URiM individuals in full‐time faculty. Further research is certainly required to explore the factors that contribute to these disparities, with focus on medical student and resident physician demographics, location of practice, and faculty with intersectional identities.

## FUNDING INFORMATION

This research received no specific grant from any funding agency in the public, commercial, or not‐for‐profit sectors. Dr. Faisal Khosa is the recipient of the Michael Smith Health Research BC Health Professional‐Investigator award (2023–2028); Don Rix Physician Leadership Lifetime Achievement Award (2022); BC Achievement Foundation – Mitchell Award of Distinction (2022); University of British Columbia – Distinguished Achievement Award for Equity, Diversity & Inclusion (2022) and Vancouver Medical Dental & Allied Staff Association – Equity, Diversity & Inclusion Award (2022).

## DISCLOSURE

The authors declare no potential conflict of interest.
